# Bilateral dentigerous cysts and ectopic teeth in the maxillary sinuses: A case report and literature review

**DOI:** 10.1016/j.ijscr.2019.01.012

**Published:** 2019-01-29

**Authors:** Badria AlKhudair, Abdulrahman AlKhatib, Ghaleb AlAzzeh, Ali AlMomen

**Affiliations:** a College of Medicine, Imam Abdulrahman Bin Faisal University, Khobar, Saudi Arabia; b ENT Specialist At King Fahad Specialist Hospital, Dammam, Saudi Arabia; c Consultant ENT, Rhinology and Endoscopic Skull Base Surgery at King Fahad Specialist Hospital, Dammam, Saudi Arabia

**Keywords:** Dentigerous cyst, Ectopic teeth, Maxillary sinus, Recurrent sinusitis, Case report

## Abstract

**Introduction:**

Dentigerous cysts are the second most common form of benign odontogenic cysts. These cysts are commonly single lesions. Bilateral and multiple dentigerous cysts are very rare.

**Case presentation:**

This article presents a rare case report of bilateral dentigerous cysts associated with two ectopic teeth located atypically in the maxillary sinuses in a 19-year-old male manifesting as bilateral upper jaw pain, post-nasal discharge and recurrent sinusitis which was managed via the endonasal endoscopic approach by enucleation of the cysts and teeth removal.

**Conclusion:**

This paper presented our experience in the management of a rare case of bilateral dentigerous cysts associated with ectopic teeth in the maxillary sinuses. Ct scan of the paranasal sinuses confirmed the diagnosis and the endonasal endoscopic approach is superior to the external approaches in preserving physiological function in managing these patients.

## Highlights

• Dentigerous cysts are commonly single lesions. Bilateral and multiple dentigerous cysts are very rare.
• Ectopic tooth eruption outside the oral cavity is a rare entity.
• The endonasal endoscopic approach has the advantages of avoiding external mucosal incision, oroantral fistula formation and recurrent sinusitis.

## 1 Introduction

Dentigerous cyst, also known as follicular cyst is the second most common form of benign developmental odontogenic cysts that results from accumulation of fluid between reduced enamel epithelium and the crown of an unerupted tooth [[1], [2], [3]]. The most frequently involved tooth is the mandibular third molar followed by the maxillary canine, but they may also be associated with supernumerary or ectopic tooth [1,4]. However, the involved tooth may be greatly displaced into ectopic positions. In the maxilla, these teeth are often displaced into the maxillary sinus [4]. The dentigerous cyst progress slowly and may pass unnoticed for several years. When the maxillary sinus is invaded by a cyst and ectopic tooth, symptoms usually occur late in process [2].

Odontogenesis occurs by means of a complicated interaction between the oral epithelium and the underlying mesenchymal tissue. Abnormal tissue interaction during this process can result in ectopic tooth development. Ectopic tooth eruption may result owing to one of 3 processes: pathological process, such as tumor or cyst, developmental disturbance, or iatrogenic activity [5]. Although it is commonly encountered dental problem, an ectopic eruption outside the oral cavity is a rare entity [4].

The standard management of the dentigerous cyst is enucleation and extraction of the impacted tooth associated with it [6,7].

To our knowledge, we believe that our case is one of the only 5 cases to present bilateral dentigerous cysts and ectopic teeth in the maxillary sinuses.

The case has been reported in line with the SCARE criteria [8]. Informed consent has been obtained from the patient for publication of this case report.

## 2 Case report

A 19-year-old Saudi male was referred to the Department of ENT at our institution with the complaint of facial pain over the upper jaw area along with post-nasal discharge. This complaint has developed over a period of 6 months prior to his presentation. The patient gave a history of recurrent sinusitis but had no other systemic illness, no past surgical history and no history of trauma. No known drug history, no family history of any genetic disorder. The patient and both parents are non-smokers.

**Fig. 1:**
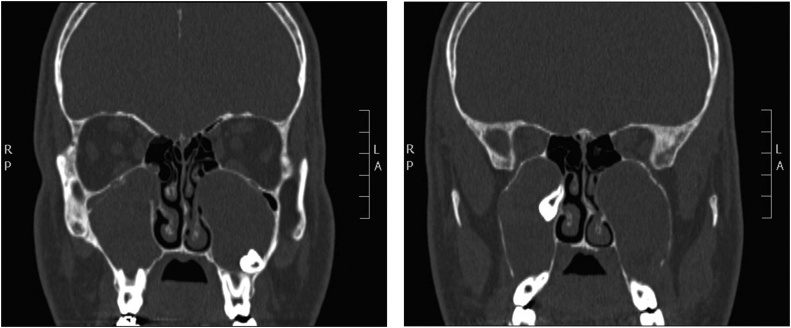
Coronal CT sinuses showing bilateral cystic lesions in the maxillary antra with expansion and remodeling of the roof, lateral and medial walls. The right cystic lesion displaces ectopic tooth superiorly and medially in the region of the expanded remodeled osteomeatal complex. The left ectopic tooth is impacted in the inferior portion of the lateral wall.

Endoscopic examination was unremarkable except for a septal spur to the left side. Paranasal sinuses computed tomography (CT) scan showed bilateral cystic lesions and ectopic teeth in both maxillary sinuses (Fig. 1).

The patient was booked for endonasal endoscopic enucleation of the cysts and extraction of the ectopic impacted teeth.

Intra-operative, bilateral big cystic masses completely filling both maxillary sinuses were visualized along with a tooth impacted in the floor of the left maxillary sinus and another tooth identified within the right osteomeatal complex obstructing the right maxillary ostium.

**Fig. 2:**
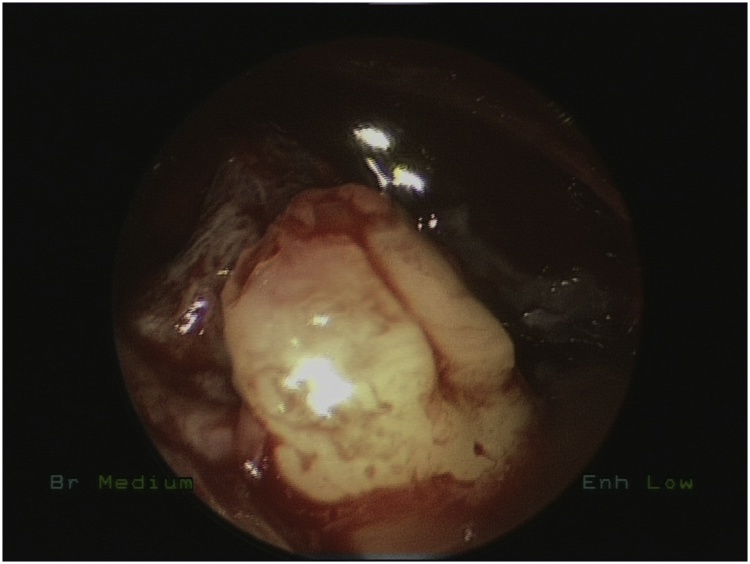
Intraoperative Endoscopic Maxillary antrostomy showing the ectopic tooth.

**Fig. 3:**
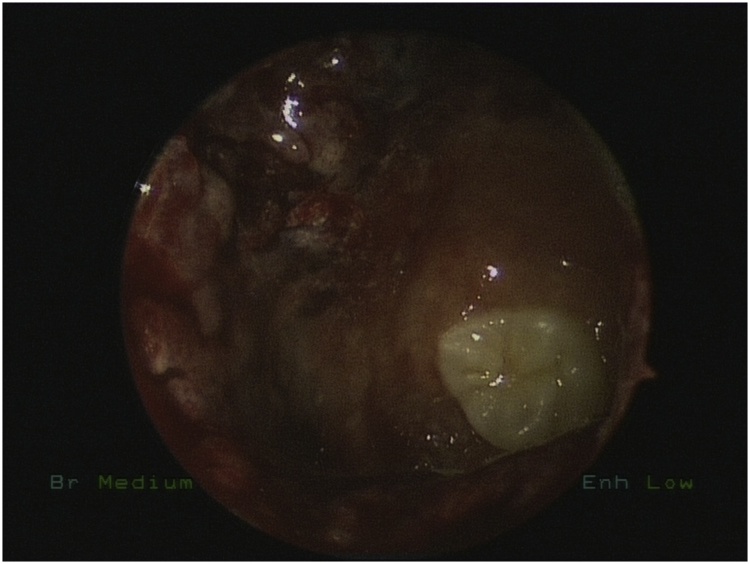
angled endoscopic view showing the tooth impacted in the floor of the left maxillary sinus.

**Fig. 4:**
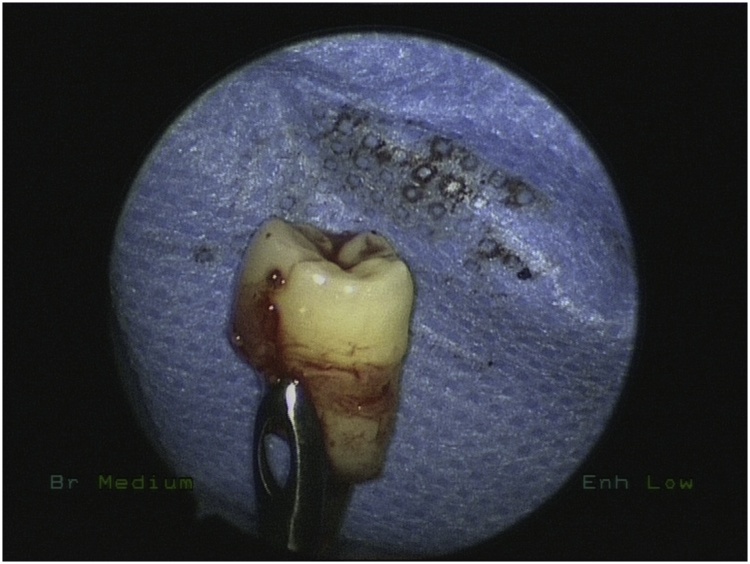
tooth removed by giraffe forceps.

Bilateral endoscopic wide middle meatal antrostomies were performed under general anesthesia. The cystic masses were dissected from the wall of both maxillary sinuses and removed by using different angel forceps and endoscopes. The right tooth was obstructing the maxillary sinus drainage (Fig. 2) removed with the cyst while the left was impacted in the left inferiolateral walls of left maxillary sinus (Fig. 3) removed completely with angled giraffe forceps (Fig. 4). Homeostasis was achieved in both sinuses and no nasal packing was needed.

The specimen was sent for histopathologic examination which confirmed the diagnosis of dentigerous cysts.

The patient’s symptoms were resolved completely post-operatively and remained free of symptoms for 5 years follow up.

## 3 Discussion

Eruption of ectopic teeth into regions outside the oral cavity is rarely reported in the literature. Although there have been reports of teeth in various regions of jaws such as mandibular condyle, coronoid process, nasal septum, palate and maxillary sinus [9]. Ectopic teeth mostly have no symptoms. However, when they present in the maxillary sinus this can cause several symptoms, most frequently a facial pain, facial swelling, headache, purulent discharge, recurrent sinusitis and nasal obstruction [4,10]. In our case, the chief complaint was facial pain involving the area of the upper jaw along with post-nasal discharge and recurrent sinusitis.

Dentigerous cysts account for 24% of all jaw cysts. They are the second most prevalent type of odontogenic cysts after periapical or radicular cysts. In 70% of the cases they develop in the mandible, only about 30% of the cases occurs in the maxilla. They usually present in the second or third decade of life with no gender predisposition [6,11]. The incidence of impacted teeth that undergo DC transformation is nearly 1.44% [12]. Dentigerous cysts related to ectopic teeth are quite rare to occur within the maxillary sinus [4].

These cysts are commonly single lesions. Bilateral and multiple dentigerous cysts are very rare although they have been reported in patients with syndromes such as basal cell nevus syndrome, mucopolysaccharidosis, and cleidocranial [12]. Bilateral maxillary dentigerous cysts in non-syndromic patients are even more rare. To our knowledge, our case is the fifth case to be reported in the literature [13].

Dentigerous cysts are generally painless. Nevertheless, delayed tooth eruption or facial swelling may be the main complain [6]. When the maxillary sinus is invaded, classic symptoms of sinus disease such as facial pain, headache, purulent nasal discharge or nasolacrimal obstruction may occur [4]. They are often discovered incidentally through routine radiological examination [9].

On radiographic examination, dentigerous cysts appear as unilocular lucent cysts of varying sizes, with well-defined sclerotic borders, associated with the crown of an unerupted tooth. If a follicular space on radiography is more than 5 mm, an odontogenic cyst can be suspected [14]. Other odontogenic cysts like radicular cysts, odontogenic keratocysts, and odontogenic tumors such as ameloblastoma, Pindborg tumor, odon- toma, odontogenic fibroma, and cemetomas may share the same radiologic features as dentigerous cysts [15,16].

The standard treatment for a dentigerous cyst in the maxillary sinus is enucleation and extraction of the impacted tooth via a Caldwell-Luc procedure [4]. The endonasal endoscopic approach for its management is also described in the literature, which was followed in our case. The endonasal endoscopic approach is a minimally invasive approach, with the advantages of avoiding external mucosal incision, oroantral fistula formation and recurrent sinusitis. The prognosis of such condition is excellent with no recurrence.

## 4 Conclusion

This paper presented our experience in the management of a rare case of bilateral dentigerous cysts associated with ectopic teeth in the maxillary sinuses. Ct scan of the paranasal sinuses confirmed the diagnosis and the endonasal endoscopic approach is superior to the external approaches in managing these patients.

## Conflicts of interest

The authors declare that there is no conflict of interest regarding the publication of this paper.

## Sources of funding

There is no financial support and sponsorship.

## Ethical approval

According to our institution guideline, case report doesn’t require ethical approval.

## Consent

Written informed consent was obtained from the patient for publication of this case report and any accompanying images.

## Registration of research studies

None.

## Guarantor

Dr. Ali Almomen.

## Data availability

The data used to support the findings of this study are included within the article. Also, they are available from the corresponding author upon request.

## Methods

This work has been reported in line with the SCARE criteria.

## Provenance and peer review

Not commissioned, externally peer-reviewed
